# Identification of male-fertility gene *AsaNRF1* and molecular marker development in cultivated garlic (*Allium sativum* L.)

**DOI:** 10.3389/fpls.2024.1419260

**Published:** 2024-05-28

**Authors:** Zezhou Liu, Naibin Duan, Zonghui Yang, Lixin Yue, Zhangjun Fei, Suping Kong

**Affiliations:** ^1^ Institute of Vegetables, Shandong Academy of Agricultural Sciences/Key Laboratory for Biology of Greenhouse Vegetables of Shandong Province/National Center for Vegetable Improvement, Jinan, China; ^2^ Institute of Crop Germplasm Resources, Shandong Academy of Agricultural Sciences, Jinan, China; ^3^ Boyce Thompson Institute, Cornell University, Ithaca, NY, United States

**Keywords:** garlic, male-fertility, callose, transcriptome, molecular marker

## Abstract

Garlic cultivars are predominantly characterized by their sterility and reliance on asexual reproduction, which have traditionally prevented the use of hybrid breeding for cultivar improvement in garlic. Our investigation has revealed a notable exception in the garlic line G398, which demonstrates the ability to produce fertile pollen. Notably, at the seventh stage of anther development, callose degradation in the sterile line G390 was impeded, while G398 exhibited normal callose degradation. Transcriptome profiling revealed an enhanced expression of the callose-degrading gene, *AsaNRF1*, in the mature flower buds of the fertile line G398 compared to the sterile line G390. An insertion in the promoter of *AsaNRF1* in G390 was identified, which led to its reduced expression at the tetrad stage and consequently delayed callose degradation, potentially resulting in the male sterility of G390. A discriminatory marker was developed to distinguish between fertile G398 and sterile G390, facilitating the assessment of male fertility in garlic germplasm resources. This study introduces a practical approach to harnessing garlic hybridization, which can further facilitate the breeding of new cultivars and the creation of novel male-fertile garlic germplasm using modern molecular biology methods.

## Introduction

Garlic (*Allium sativum* L.) belongs to the genus *Allium* in the Alliaceae family and is widely grown around the world. Garlic cultivars cannot produce fertile pollen, nor do they produce seeds (Kamenetsky, 2007), and can be reproduced only vegetatively through bulblets and cloves (Novák and Havránek, 1975; Shemesh-Mayer and Kamenetsky-Goldstein, 2021). This mode of reproduction hinders the introduction of new genetic variations through sexual crosses and the selection of superior cultivars from variable seedling populations. Male gametogenesis and sterility are barriers to fertilization and seed production in garlic (Shemesh Mayer et al., 2013). Flowering and seed production have been reported in wild garlic (*A. longicuspis*) from Central Asia (Pooler and Simon, 1994; Kamenetsky and Rabinowitch, 2001; Kamenetsky et al., 2005; Lee et al., 2015), and true garlic seeds have been produced (Jenderek and Zewdie, 2005; Shemesh et al., 2008). However, most of the collected fertile garlic genotypes still have poor fertility or do not produce reproductive organs at all (Shemesh-Mayer and Kamenetsky-Goldstein, 2021). Identification of garlic germplasm with suitable reproductive potential is still quite challenging today (Shemesh-Mayer and Kamenetsky-Goldstein, 2021).

Plants with male sterility cannot produce fertile pollen, and this male sterility may be regulated by genes encoded in the nuclear (genic male sterility) or mitochondrial (cytoplasmic male sterility) genomes (Garcia et al., 2019). Abnormalities in the formation of the pollen sac (Chaban et al., 2020; Zheng et al., 2021), meiosis (Qiu et al., 2018; Shin et al., 2021), callose (Pu et al., 2019; Li et al., 2020), tapetum (Mondol et al., 2020; Zhang et al., 2021), pollen wall (Ren et al., 2020; Deng et al., 2022), and anther dehiscence (Kim and Kim, 2021; Qi et al., 2022) during pollen development can lead to pollen abortion and a male-sterile phenotype. Tapetum and meiocytes are primarily affected in most sporophytic male-sterile mutants (Guo and Liu, 2012). In contrast, the development of microspores or pollen grains is mainly affected in gametophytic male-sterile mutants (Chen and Liu, 2014). The tapetum serves mostly as a meiocyte/spore nutrition tissue commonly found in terrestrial plants (Pacini et al., 1985). Normal tapetum function requires the properly timed initiation and progression of programmed cell death (PCD) (Papini et al., 1999; Kawanabe et al., 2006; Ji et al., 2013). Non-regulated PCD of the tapetum can lead to energy deficiency and consequent pollen abortion in garlic (Shemesh-Mayer et al., 2015). Abnormal tapetal development has been previously described as a possible cause of male sterility in garlic (Novak, 1972; Shemesh-Mayer et al., 2015), onion (*Allium cepa*) (Holford et al., 1991), lily (*Lilium Asiatic*) (Wang et al., 2019) and *Arabidopsis thaliana* (Chaudhury et al., 1994).

Callose is a linear 1,3-β-glucan polymer that is widespread in cell walls of higher plants (Stone and Clarke, 1992). It is also a major component of the tetrad wall, which is wrapped around four microspores formed by meiosis of the microsporocytes (Francis and Copenhaver, 2006). The development of pollen is affected by abnormal callose or tapetum degradation, which is one of the main causes of male sterility in plants. Callose is synthesized by glucan synthase-like (GSL) and degraded by the enzyme endo-1,3-β-glucosidase (Shi et al., 2016; Pu et al., 2019). The amount of callose gradually increases during meiosis but decreases at the end of microsporogenesis due to the activity of callase (Stieglitz, 1977; Chen and Kim, 2009). A lack of callose, premature disintegration, or abnormal deposition, causes plants to produce sterile pollen (Abad et al., 1995; Wan et al., 2011). The abnormal degradation of callose walls is probably the primary cause of the appearance of cytoplasmic male-sterile lines in petunia, sorghum, soybean (Leubner-Metzger and Meins, 1999), and *Arabidopsis* (Fei and Sawhney, 1999). The post-meiotic release of microspores from the common callose wall is strictly conditioned by the activity of callase (McCormick, 2004; Mamun et al., 2005). Deficiency in the activity of callosinase that degrades the callose wall can cause male sterility in rice (Wan et al., 2011). Töller et al (Töller et al., 2008). showed that in the *Arabidopsis gsl10* mutant, persistence of the callose wall may interfere with the migration of germ cells, possibly leading to the generative cell being tightly stuck against the wall later in development. Callose surrounding tetrads cannot be degraded over time, and abnormal callose deposition is observed during the abortion of microspores in nuclear sterile Chinese cabbage near-isogenic line ‘10L03’ (Pu et al., 2019). Callose degradation is delayed in the *res3 Arabidopsis* male-sterile mutant; however, delayed callose degradation restores the fertility of *rvms-2* (Wang et al., 2021). Another possibility is that the tapetum is not fully functional and cannot synthesize callase, and thus the callose wall is not degraded (Fei and Sawhney, 1999). Through research on the anthers of sterile *Allium sativum* and fertile *Allium atropurpureum*, Winiarczyk et al (Winiarczyk et al., 2012). found that the activity of callase (*β*-1,3-β-glucanase) during microsporogenesis may regulate the degradation of the callose wall and indirectly affect male gametophyte development. To date, the causes of garlic floral abortion and sterility are still not well understood (Simon and Jenderek, 2004; Shemesh-Mayer and Kamenetsky-Goldstein, 2021).

Cultivated garlic is a natural male-sterile material and an ideal female parent for hybrid production. Limited by the availability of male-fertile garlic cultivars, research has mainly focused on the analysis of organ- and tissue-specific gene expression in garlic (*Allium sativum* L.) using transcriptome data (Kamenetsky et al., 2015; Shemesh-Mayer et al., 2015; Havey and Ahn, 2016). The release of the garlic genome (Sun et al., 2020; Hao et al., 2023), and especially the availability of a fertile garlic cultivar G398, which has been identified and maintained by our group for more than 20 years, provide a strong foundation for a systematic analysis of the molecular mechanisms underlying the sterility of garlic cultivars.

## Materials and methods

### Plant materials

Two garlic cultivars, namely G390 (a male-sterile garlic cultivar) and G398 (a male-fertile garlic cultivar), were used in this study. Both garlic lines were planted at the experimental site of the Vegetable Research Institute, Shandong Academy of Agricultural Sciences, and grown under natural conditions. They were planted in October of the first year and harvested in June of the following year. The temperature during the growing period ranged from -10°C to 35°C. The photoperiod ranged from 10-14 hours of daylight to 14-10 hours of night.

### Phenotypic and microscopic studies of garlic flowers

After the garlic plants had bolted, to enhance flower bud growth, the aerial bulbs were removed. Under both fertile and sterile conditions, flower buds and inflorescences at different developmental stages were selectively collected based on their respective bud lengths. To facilitate microscopic examination, paraffin sectioning was then performed. First, formalin-acetic acid-alcohol (FAA) fixative (5% formaldehyde, 45% alcohol, and 5% acetic acid) was used to fix the collected samples. Secondly, the samples were rehydrated in two changes of BioDewax and Clear Solution and 100% - 100% - 75% alcohol. Each step takes anywhere for 5 minutes, rinse with running water. Thirdly, put sections into safranin-O-staining solution for 15-30 seconds s and three cylinders of anhydrous ethanol for rapid dehydration. Fourthly, put slides into 50%, 70% and 80% alcohol for 3-8 seconds. Fifthly, sections into plant solid green staining solution staining 6-20 seconds, anhydrous ethanol three-cylinder dehydration. Sixthly, put sections into three cylinders of xylene for 5 minutes, mount the tissue section with neutral balsam. Finally, Observed under microscope and took images. These technical processes were performed by Wuhan Servicebio Technology Co., Ltd (Wuhan, Hubei, China). The stain used in this study was Safranin O-Fast Green staining solution (Servicebio, G1031).

Alexander stain was used to observe the viability of garlic pollen, and the test procedure was carried out according to the manufacturer’s instructions.

### Flower bud collection and RNA extraction

The early inflorescences (EFs) of the two garlic lines were carefully sampled when the inflorescences of garlic sprouts emerged approximately 2-3 cm above the ground. Subsequently, as the garlic shoot expanded its pseudostem and the inflorescence dissolved, flower buds of 2.5-3.5 mm in length (tetrad-stage flower buds, TFs) were collected. Each sample consisted of three independent biological replicates, each of which contained flower buds from 10 individual plants. All collected samples were immediately frozen in liquid nitrogen and stored at -80°C till the RNA extraction. Trizol reagent (Invitrogen) was used to perform RNA extraction, followed by treatment with RNase-free DNase I (TaKaRa) in strict compliance with the protocols provided by the respective manufacturers. Prior to transcriptome sequencing, the RNA purity, concentration, and integrity of each sample were carefully assessed.

### Library preparation and sequencing

Magnetic Oligo (dT) Beads (Illumina) were used to achieve the enrichment of poly (A) mRNA from 20 µg of total RNA samples, followed by fragmentation of the enriched mRNA into shorter fragments using Ambion’s RNA kit prior to cDNA synthesis. Short mRNA fragments were used as templates with random primers and reverse transcriptase (Invitrogen) for first-strand cDNA synthesis. Subsequently, DNA polymerase I was used to synthesize second-strand cDNA. RNase H was used to eliminate the RNA template from double-stranded cDNAs. AMPure XP beads were used to purify the resulting double-stranded cDNAs, following the manufacturer’s recommended protocol. An Agilent 2100 Bioanalyzer (Krupp, 2005) was used to assess the insert sizes, specifically 500 bp, of the constructed RNA-Seq libraries. Ultimately, the library products were sequenced at the Beijing Genomic Institute using the BGISEQ-500 system with a paired-end 125-bp mode (Fu-Yuan et al., 2018).

### RNA-Seq read processing and DEG identification

To remove the adaptor and low-quality sequences, raw RNA-Seq reads were cleaned using Trimmomatic 0.38 (Bolger et al., 2014). Reads shorter than 40 bp were discarded. Trimmed reads were then aligned to the ribosomal RNA database (Quast et al., 2013) using Bowtie 2.0 (Langmead et al., 2009) allowing up to three mismatches, and only unmapped reads were kept for downstream analysis. Furthermore, the cleaned, high-quality paired-end reads were mapped to the garlic reference genome (Sun et al., 2020) using HISAT2 (Kim et al., 2019) (version 2.1.0) with default parameters. Based on the alignments, the counts of mapped reads for each gene were calculated and normalized to the FPKM values. DEGs between G398 and G390 and between the two developmental stages (EF and TF) were identified using DESeq2 (Love et al., 2014) with the following criteria: fold change ≥ 2 and adjusted p value < 0.05. GO term enrichment analysis was conducted using the R package ClusterProfiler (Wu et al., 2021), and KEGG pathway enrichment analysis was carried out through the KOBAS online service (http://bioinfo.org/kobas) (Dechao et al., 2021). TBtools (Chen et al., 2020) was used to plot gene expression heatmaps.

### Gene amplification and InDel marker verification

The ApexHF HS DNA Polymerase FS Master Mix (Accurate Biotechnology (Hunan) Co., Ltd.) was used for PCR amplification of genomic sequences following the manufacturer’s instructions. The genomic DNA extracted from the fertile line G398 and the sterile line G390 served as PCR templates, and the resulting amplicons were subsequently subjected to agarose gel electrophoresis.

Sanger sequencing of the amplified products was performed at Shangon Biotech and BioSune. Regarding the InDel marker, standard PCR polymerase was used for amplification. Subsequently, the PCR products were subjected to electrophoresis using a 1.5% agarose gel, separated under a constant power of 140 V for 1 hour, followed by staining with 0.5 μg/mL ethidium bromide in a 1× TBE solution for 15 minutes. After rinsing with water, to facilitate further band pattern statistics, the gel was photographed using an ultraviolet gel imaging system.

## Results

### Delayed callose degradation during anther development in male-sterile garlic cultivar G390

**Figure 1:**
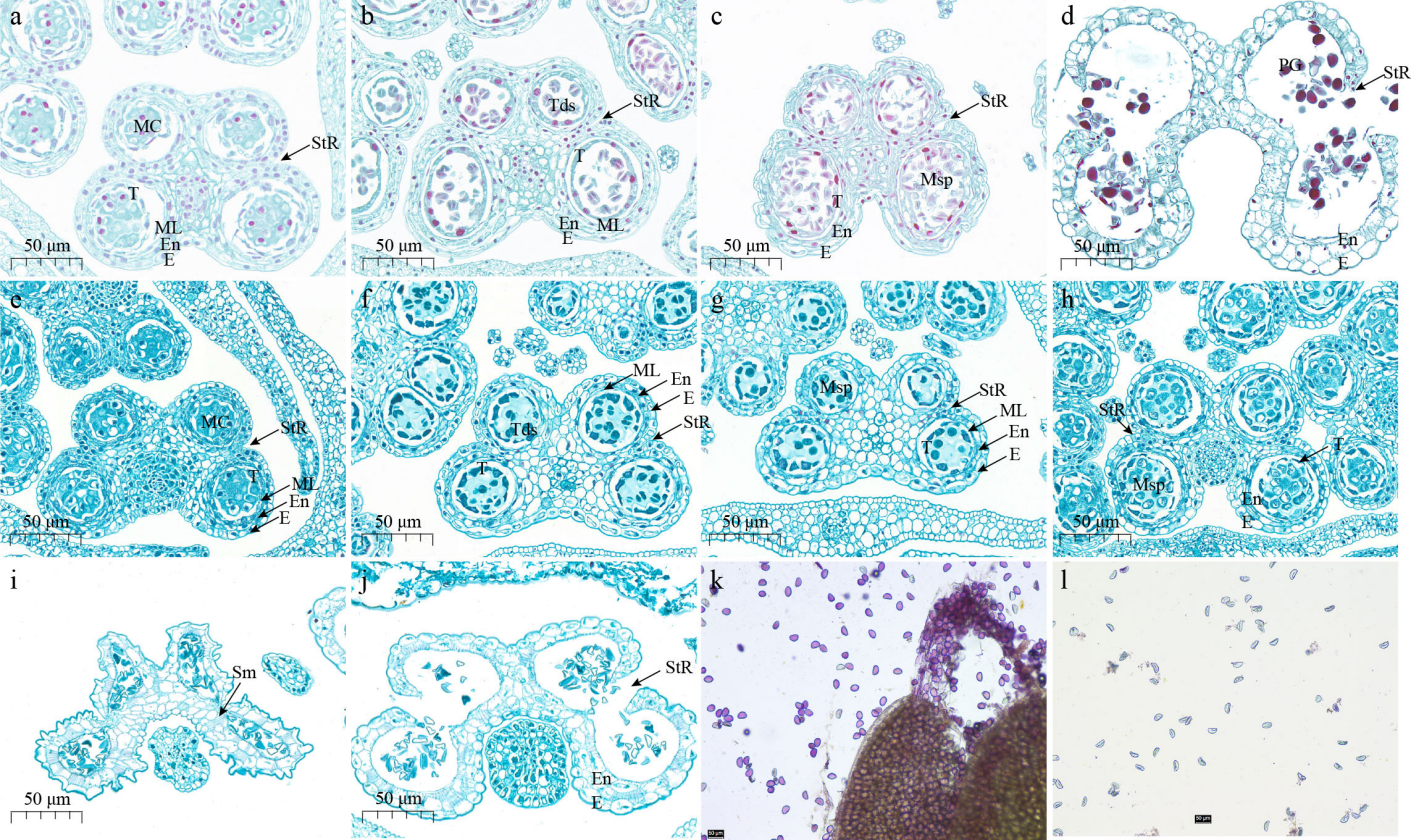
Anther paraffin sections and pollen staining of fertile line G398 and sterile line G390. **(A–D)**, Anther paraffin sections of the fertile line G398 at stages 6 **(A)**, 7 **(B)**, 8 **(C)** and 13 **(D)**. **(E–J)**, Anther paraffin sections of the sterile line G390 at stages 6 **(E)**, 7 **(F)**, 8 **(G)**, 9 **(H)** and 13 **(I, J)**. Stage 6: Microspore mother cells enter meiosis. Middle layer is crushed and degenerates. Tapetum becomes vacuolated and the anther undergoes a general increase in size. Stage 7: Meiosis is completed. Tetrads of micropsores are free within each locule. Remnants of middle layer are present. Stage 8: Callose wall surrounding tetrads degenerates and individual microspores are released. Stage 9: Growth and expansion of anther continue. Microspores generate an exine wall and become vacuolated. Stage 13: Dehiscence (Sanders et al., 1999). E, epidermis; En, endothecium; MC, meiotic cell; ML, middle layer; MSp, microspores; PG, pollen grains; Sm, septum; St, stomium; StR, stomium region; T, tapetum; Tds, tetrads. **(K)** Pollen staining of G398. **(L)** pollen staining of G390. Bar =50 µm.

Paraffin sections of the fertile line G398 and the sterile line G390 are illustrated in **Figure 1**. According to the 14 stages of anther development in *Arabidopsis thaliana* (Sanders et al., 1999), the anther development of G398 and G390 showed no significant difference at the sporogenous cell stage and the pollen mother cell stage. Both lines formed normal dyads and tetrads, and obvious callose was observed during stage 6 of anther development (**Figures 1A, E**). In G398, callose degradation was observed during stages 7 and 8 of anther development (**Figures 1B, C**), and microspores developed normally. However, in G390, callose was not degraded at stage 7 and still existed till stage 9 of anther development, and microspore development was abnormal (**Figures 1F–H**). At stage 13 of anther development, the anther chamber of G398 cracked and released mature pollen grains (**Figure 1D**), while the anther chamber of G390 could also crack and release pollen, but the anther chamber was twisted and deformed, and the pollen grains were mostly deformed and gathered into clusters (**Figures 1I, J**). Alexander staining showed that the pollen of G398 was vigorous, while the pollen of G390 was inactive (**Figures 1K, L**). In general, at the early stages meiosis was normal in both genotypes, but the delayed callose degradation in the sterile line G390 may lead to its male-sterile phenotype after the formation of tetrads in stage 7 of anther development.

### Transcriptome sequencing and analysis of floral development

Due to the delay in callose degradation of the sterile line G390, RNA-Seq was performed on the early inflorescence (EF) and tetrad-stage flower buds (TF) of G390 and G398. Around 35.1 million raw read pairs and 33.7 million cleaned read pairs were obtained for each sample.

**Figure 2:**
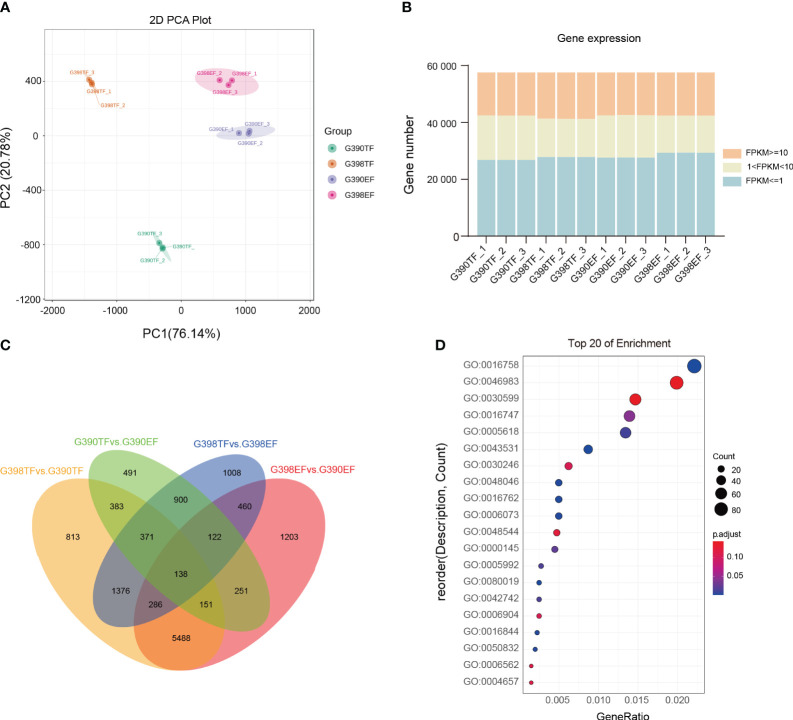
Transcriptomic analysis of the fertile line G398 and the sterile line G390. **(A)** PCA of the RNA-Seq data. Each color represents a group of biological replicates from the same sample. **(B)** Numbers of detected genes in each sample. Different colors represent different ranges of FPKM values. **(C)** Venn diagram showing numbers of DEGs identified between different samples; **(D)** Top 20 GO terms enriched in DEGs between G398TF and G390TF.

The cleaned reads were aligned to the garlic genome (Sun et al., 2020), with alignment rates ranging between 84.87% and 92.36% (**Supplementary Table S1**). Principal component analysis (PCA) suggested high correlations between the biological replicates of each sample (**Figure 2A**). Based on the alignments, counts of mapped reads for each garlic gene were calculated and then normalized to the fragments per kilobase of transcripts per million mapped fragments (FPKM) values. Genes with normalized expression levels above 1.0 FPKM were used for the downstream analyses. Approximately 24.75% of the expressed genes had FPKM values of 1–10, and 26.77% ≥10 FPKM (**Figure 2B**, **Supplementary Table S2**). Overall, we concluded that the quality and depth of the RNA-Seq data were sufficient for our downstream analyses.

Differentially expressed genes (DEGs) in different comparisons were identified. Among the 13,441 identified DEGs, 138 were common in all four comparisons: G398 TF vs. G390 TF, G390 TF vs. G390 EF, G398 TF vs. G398 EF, and G398 EF vs. G390 EF. A total of 813 DEGs were specifically identified in the G398 TF vs. G390 TF comparison, 491 were specifically identified in the G390 TF vs. G390 EF comparison, 1008 in the G398TF vs. G398EF comparison, and 1203 in the G398EF vs. G390EF comparison (**Figure 2C**).

We then identified gene ontology (GO) terms enriched in DEGs between G398 TF and G390 TF. The enriched GO terms were related to a range of biological processes including recognition of pollen (GO:0048544), cellular glucan metabolic process (GO:0006073) and vesicle docking during exocytosis (GO:0006904), molecular functions including hexosyltransferase activity (GO:0016758), acyltransferase activity, transferring groups other than amino-acyl groups (GO:0016747), xyloglucosyl transferase activity (GO:0016762), pectinesterase activity (GO:0030599), carbohydrate binding (GO:0030246), alcohol-forming very long-chain fatty acyl-CoA reductase activity (GO:0080019) and strictosidine synthase activity (GO:0016844), and cellular components including cell wall (GO:0005618) (**Figure 2D**). These results provide molecular insights into male sterility, illustrating how variations in cell wall metabolism and related processes between G398 and G390 contribute to the observed phenotypic differences.

**Figure 3:**
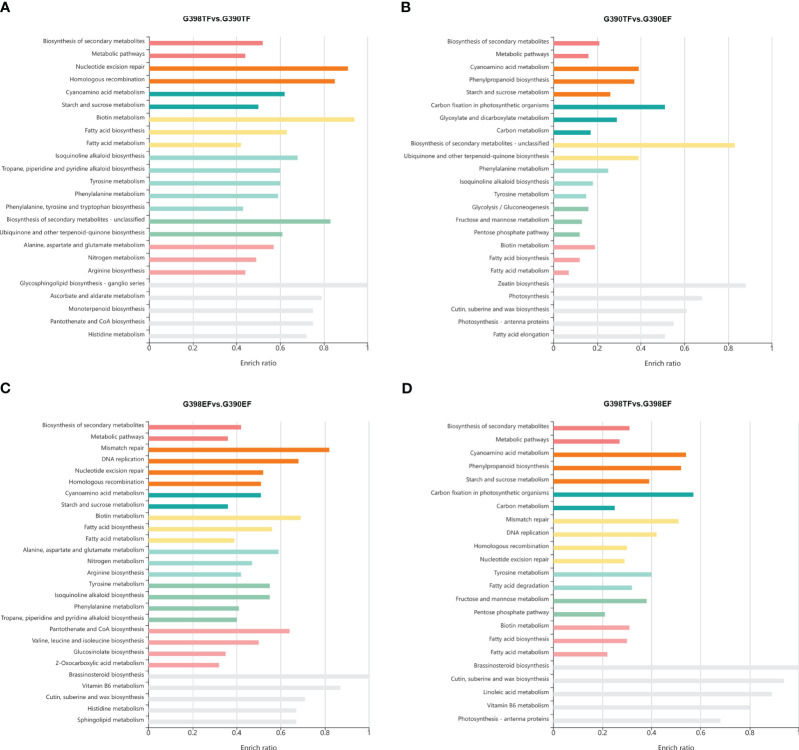
KEGG enrichment analysis of DEGs between the fertile line G398 and the sterile line G390. a-d, KEGG enrichment analysis of DEGs in TF between G398 and G390 **(A)**, in G390 between EF and TF **(B)**, in EF between G398 and G390 **(C)**, and in G398 between TF and EF **(D)**.

Kyoto Encyclopedia of Genes and Genomes (KEGG) (Kanehisa and Goto, 2000) enrichment analysis revealed that the biosynthesis of secondary metabolites, metabolic pathways, nucleotide excision repair, homologous recombination, cyanoamino acid metabolism, and starch and sucrose metabolism pathways were enriched in the DEGs between G398 TF and G390 TF (**Figure 3A**); biosynthesis of secondary metabolites, metabolic pathways, mismatch repair, DNA replication, nucleotide excision repair, and homologous recombination pathways in DEGs between G398 EF and G390 EF (**Figure 3C**); and biosynthesis of secondary metabolites, metabolic pathways, cyanoamino acid metabolism, phenylpropanoid biosynthesis, starch and sucrose metabolism, and carbon fixation in photosynthetic organisms in DEGs between the EF and TF of G390 and G398 (**Figures 3B, D**). K-means clustering analysis of DEGs identified five clusters, including one containing 192 genes that were specific highly preferentially expressed in the tetrad-stage flower buds of the fertile line G398 (**Supplementary Figure S1E**, **Supplementary Table S3**). These 192 genes, highly expressed in the tetrad-stage flower buds of the fertile line G398, might play a pivotal role in shaping the intricate processes of anther development and flower maturation. Future experiments are required to characterize the functions and interactions of these genes, which will offer valuable insights into the mechanisms underlying sterility and the intricacies of garlic flower development.

### Genes related to pollen development and callose synthesis and degradation

**Figure 4:**
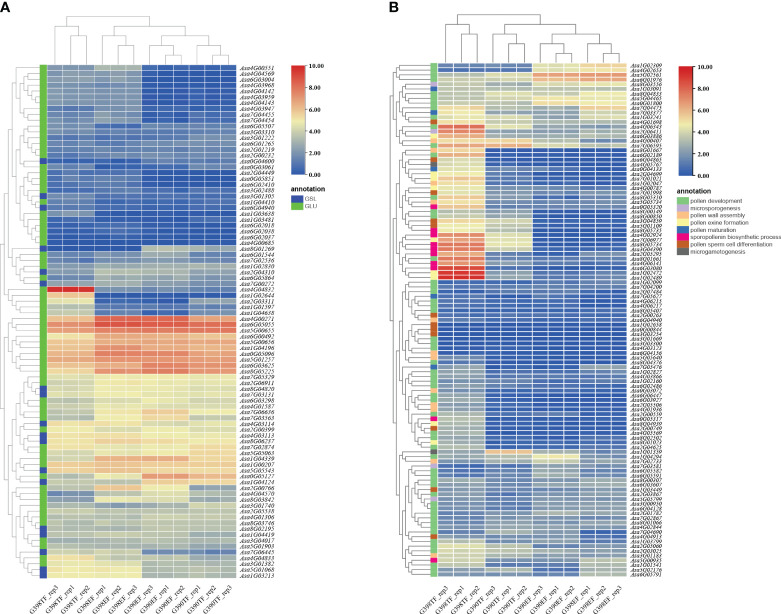
Heatmap of the expression of GSL, GLU and pollen development-related genes in garlic. **(A)** Expression of GSL and GLU homologous genes in the fertile line G398 and the sterile line G390. **(B)** Expression of pollen development-related genes in the fertile line G398 and the sterile line G390.

To search for genes related to garlic pollen development and genes involved in callose synthesis and degradation, amino acid sequences of 353 pollen development-related genes, 12 callose synthesis genes (GSLs), and 76 endo-1,3-β-glucosidases (GLUs) from *Arabidopsis thaliana* (https://www.arabidopsis.org) were used as queries to search against the protein sequences in the garlic genome using the NCBI local blast tool 2.6.0+ (Zhang et al., 2000). By selecting the top 3 homologous sequences with the highest identity, a total of 539 pollen development-related genes, 10 callose synthesis genes, and 52 callose degradation genes (**Supplementary Table S4**) were obtained. Compared with the GSL genes, the GLU genes were more differentially expressed between these two accessions and between the two flower development stages (**Figure 4A**). For example, the expression levels of *Asa6G03004*, *Asa4G04569*, *Asa4G03959*, and *Asa4G04142* were higher in the fertile line G398 than in the sterile line G390, while the expression levels of *Asa0G05127* and *Asa8G01269* were higher in G390 than in G398. Gene expression also differed among flower development stages. *Asa1G01597*, *Asa1G03638*, *Asa4G04832*, *Asa1G02644*, *Asa2G03311*, *Asa1G04410*, *Asa1G04638*, and *Asa6G04940* showed peak transcript levels in the TF of G398 whereas *Asa3G01740* and *Asa3G01222* showed peak transcript levels in the TF of G390, and *Asa0G03061* had higher transcript levels in the TF than in the EF of both G398 and G390 (**Figure 4A**). Similarly, a number of pollen development-related genes in garlic were differentially expressed between different flower development stages. The expression of most of these genes was higher in G398 than in G390, and the highest in the TF of G398 (**Figure 4B**). However, the expression levels of *Asa4G02653*, *Asa6G01976*, *Asa1G02309*, *Asa5G02561*, and *Asa7G03581* were higher in G390 than in G398, and *Asa7G02733* and *Asa1G04294* showed peak transcript levels in the EF of G390. *Asa1G01389* had higher transcript levels in the TF of G390, and *Asa8G04833* had higher transcript levels in the EF of G398 (**Figure 4A**). The substantial differences in the expression of flower development-related genes between G398 and G390 and between different flower development stages indicate that the fertility recovery of garlic is a complex regulatory process.

### Identification of garlic fertility-restorer gene *AsaNRF1* and marker development

**Figure 5:**
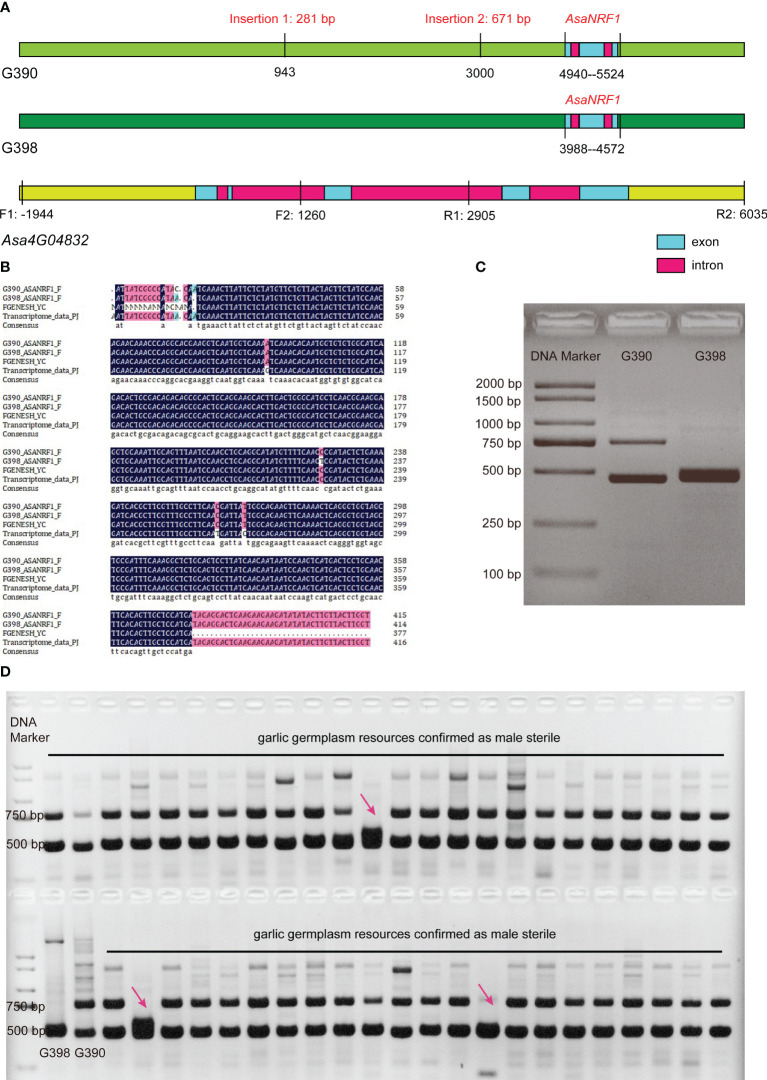
Gene structure of garlic fertility restorer gene *AsaNRF1* and development and verification of an InDel molecular marker, *AsaNRF1*-M281. **(A)** Gene structure of garlic fertility restorer gene *AsaNRF1*. Positions are relative to the *Asa4G04832* start codon ATG. F1, R1, F2 and R2 indicate relative positions of primers used to amplify the *Asa4G04832* DNA sequence. **(B)** Sequence of garlic fertility restorer gene *AsaNRF1*. **(C)** Development of the InDel molecular marker *AsaNRF1*-M281. **(D)** Verification of the InDel molecular marker *AsaNRF1*-M281.

Based on the results of the anther paraffin section, transcriptome profiling analysis, the callose degradation homologous gene *Asa4G04832*, which exhibited significant expression differences (**Supplementary Table S4**), and contained five exons and had an CDS of 1278 bp in length, was identified as the candidate male fertility-restorer gene of garlic. The full-length genomic sequence of *Asa4G04832* and its up- and downstream 2000 bp (chr4: 1,321,277,916–1,321,285,895) from these two lines were amplified (primers are listed in **Supplementary Table S5**), and Sanger sequencing was performed on the amplified products. The results showed that there were two insertions in the genome sequence of the male-sterile line G390, a 281-bp insertion at 943 bp and a 671-bp insertion at 3000 bp downstream of the start codon of *Asa4G04832* (**Figure 5A**). However, based on the RNA-Seq read mapping pattern in IGV software (Thorvaldsdóttir et al., 2013) (**Supplementary Figure S2**), the gene model of *Asa4G04832* could be wrongly predicted. Therefore, we used FGENESH 2.6 (Solovyev et al., 2006) to predict potential genes based on Sanger sequencing results, and a new gene comprising three exons and two introns, with a coding sequence of 357 bp, was predicted, which we named *AsaNRF1* (Garlic Nuclear Restoring Fertility gene 1; GenBank accession number: OR865691) (**Figure 5A**). Furthermore, this result was validated through the *de novo* assembly of transcriptome data and Sanger sequencing of cDNA (**Figures 5A, B**). Moreover, when we aligned RNA-Seq reads of G398 and G390 to the garlic genome (Sun et al., 2020; Hao et al., 2023), we identified three high coverage peaks in the *AsaNRF1* gene region, which corresponds to three predicted exons of this gene (**Supplementary Figure S3**). This discovery indicates the presence of three exons in the *AsaNRF1* gene region. NCBI amino acid sequence alignment showed that *AsaNRF1* was a homologous gene of monocot glucan endo-1,3-β-glucosidase involved in callose degradation. The 671-bp insertion in the male-sterile line G390 was located in the *AsaNRF1* promoter region, 988 bp upstream of the start codon. Thus, the insertion of the promoter region may lead to a decrease in *AsaNRF1* expression in tetrad-stage flower buds of G390, resulting in a delayed callose degradation. The normal expression and function of this gene in premature flower buds is most likely the direct cause of the male-fertile traits of the garlic line G398.

Based on the 281-bp insertion, an insertion-deletion (InDel) molecular marker, *AsaNRF1*-M281 (**Supplementary Table S5**), which could amplify approximately 728-bp and 447-bp products from the male-sterile line G390 and the fertile line G398, respectively, was developed (**Supplementary Figure S4**). Only a 447-bp amplified band existed in G398, while 728- and 447-bp amplified bands were found in G390 (**Figure 5C**). This result indicated that the fertile line G398 was shown to be homozygous at this locus, while the sterile line G390 was heterozygous, suggesting that the fertile locus was recessively inherited. A total of 46 garlic accessions, which was confirmed as male sterile through investigating their flowers, were selected to verify this marker, and 43 of them showed the bands of the sterile type; therefore, the accuracy of this marker was 93.48% (43/46) (**Figure 5D**). The above results indicate that *AsaNRF1*-M281 can be used as a molecular marker for fertility traits in garlic cultivars and can assist in the identification of the fertility traits of garlic cultivars.

## Discussion

Garlic (*Allium sativum* L.) has been widely cultivated worldwide (Lopez-Bellido et al., 2016) and has a high edible and medicinal value. Cultivated garlic is usually sterile and can only be propagated asexually by sowing bulbs or aerial bulbs (Novák and Havránek, 1975; Kamenetsky, 2007). Garlic breeding is often limited to natural or induced mutations and somaclonal variations (Shemesh-Mayer and Kamenetsky-Goldstein, 2021). Hybridization, the technique for improving cultivar characteristics, has not been applied to garlic breeding due to the scarcity of fertile garlic cultivars. Researchers have explored fertility genes and related mechanisms in the model plant *Arabidopsis thaliana* and grain crops. There are relatively few studies on fertility-related genes and regulatory networks in vegetables, and they mainly focus on staple vegetable crops, such as tomato and cucumber (Cheng et al., 2023). Through more than 20 years of research, we have screened and bred fertile garlic lines and obtained inbred and hybrid seeds (Liu et al., 2018). In this study, the first male-fertile gene, *AsaNRF1*, homologous to genes involved in callose degradation in monocots, was identified, and an InDel marker, *AsaNRF1*-M281, was developed for marker-assisted breeding.

### Delayed callose degradation during anther development in garlic leads to male sterility

Any abnormality in anther development may lead to pollen abortion and male sterility (Qiu et al., 2018; Pu et al., 2019; Chaban et al., 2020; Li et al., 2020; Mondol et al., 2020; Ren et al., 2020; Kim and Kim, 2021; Shin et al., 2021; Zhang et al., 2021; Zheng et al., 2021; Deng et al., 2022; Qi et al., 2022). The male gametophyte in garlic flowers stops at the early stage of sexual cell development (Koul and Gohil, 1970; Tchórzewska et al., 2015). The malformation of the tapetum in male-sterile garlic material MS96 is the main cause of microspore dystrophy and leads to nonviable pollen grains (Mayer et al., 2015). The orchestration of developmental PCD in the tapetum with microspore development might be regarded as the main cause of male sterility in *A. sativum* cv. Harnas and cv. Arkus (Tchórzewska et al., 2017). The period of callose wall persistence in *A*. *sativum* is the longest among all *Allium* plants (Winiarczyk et al., 2012). During microsporogenesis, the callose wall around the microspore tetrad remains intact in completely sterile garlic genotypes (Winiarczyk et al., 2012; Shemesh Mayer et al., 2013). The degradation of the callose wall around the garlic tetrad is remarkably impeded regardless of weather conditions (Winiarczyk et al., 2012). In this study, both the sterile line G390 and the fertile line G398 had normal meiosis in the early stages of anther development, but the callose degradation of G390 was delayed after the formation of tetrads at the seventh stage of anther development (**Figure 1**). In the sterile line G390, callose still existed at the ninth stage of anther development, and microspores still aggregated into clusters, which eventually led to more deformed pollen grains and inactivity. Overall, we speculate that the delay in callose degradation during anther development is the direct cause of male sterility in garlic cultivars.

### Decreased expression of *AsaNRF1* causes male sterility by delaying callose degradation in garlic cultivars

The full-length genomic sequences and 2000 bp up- and downstream regions of *Asa4G04832* in the male-fertile line G398 (7979 bp) and the male-sterile line G390 (8931 bp) were compared, which revealed insertions/deletions between these two lines. The garlic fertility-restorer gene *AsaNRF1*, a novel gene predicted from DNA sequence of *Asa4G04832* in G390 and G398, was almost not expressed in EF, but its expression in the tetrad-stage flower buds of the male-fertile line G398 was significantly higher than that of the male-sterile line G390. The 671-bp insertion in the promoter region of *AsaNRF1* in G390 may be the reason for its decreased expression. Similarly, in pepper, there was a 579-bp deletion in the upstream region of the *Up* gene, encoding an auxin transport-related protein, resulting in differential expression of this gene (Cao et al., 2022). The presence of a 1-bp deletion in the promoter region of the dominant male-sterility gene *MS-cd1* was responsible for the sterility of the dominant male-sterile mutant 79-399-3 in *Brassica oleracea* (Han et al., 2023). The homologous genes of *AsaNRF1* in *Arabidopsis* (*AT4G26830*) (Jakobsen et al., 2005) and monocots all have the glucan endo-1,3-β-glucosidase activity. Combined with the phenotype, we speculate that *AsaNRF1* may play a role in degrading callose. In summary, we hypothesize that the expression of *AsaNRF1* in the tetrad-stage flower buds is reduced due to the insertion of its promoter region, thereby losing (weakening) the function of degrading calloses and leading to the male-sterile phenotype of G390. Compared with the garlic genome (Sun et al., 2020), the nucleotide sequence of *AsaNRF1* had only a high homology with *Asa4G04832*, indicating that *AsaNRF1* is a single-copy gene. There were 16 genes with high homology (>80%) in the amino acid sequences (**Supplementary Table S6**). Transcriptome analysis showed that the expression levels of these 16 genes in the tetrad-stage flower buds of these two accessions in this study were higher than those in EF. Most of the genes had small differences in expression (fold change <2) between G398 and G390. *Asa8G00229*, *Asa4G04833*, *Asa8G04877*, and *Asa2G00766* were highly expressed in the TF of G398, two times higher than in the TF of G390, while *Asa1G04339* was highly expressed in the TF of G390, two times higher than in the TF of G398. *AsaNRF1* has many homologous genes in garlic, and the functions of these genes may overlap or be redundant. In addition, garlic transformation is very challenging and time-consuming. Therefore, in this study, there was no functional complementation of *AsaNRF1* in garlic. This gene and its regulatory mechanism of fertility recovery require further research and verification.

## Data availability statement

Raw RNA-Seq data has been deposited in the National Center for Biotechnology Information (NCBI) BioProject database under the accession number PRJNA1047198.

## Author contributions

ZL: Data curation, Formal analysis, Investigation, Writing – original draft. ND: Data curation, Methodology, Writing – review & editing. ZY: Formal analysis, Visualization, Writing – review & editing. YL: Formal Analysis, Visualization, Writing – review & editing. ZF: Methodology, Writing – review & editing. SK: Conceptualization, Project administration, Resources, Writing – review & editing.

## Supplementary material

Supplementary Figure 1 K-means clustering of differentially expressed genes. **(A)** Non-prefer-expressed DEGs between G398 and G390. **(B)** Genes both highly expressed in G398TF and G390TF (median value of foldchange < 2). **(C)** Genes that were specific highly preferentially expressed in G398. **(D)** Genes that were specific highly preferentially expressed in G390. **(E)** Genes that were specific highly preferentially expressed in the tetrad-stage flower buds of the fertile line G398.

Supplementary Figure 2 RNA-Seq read-mapping pattern of the callose degradation homologous gene *Asa4G04832*. Read coverages of *Asa4G04832* in the fertile line G398 and the sterile line G390 are shown. Positions are relative to the *Asa4G04832* start codon.

Supplementary Figure 3 RNA-Seq read-mapping pattern of *AsaNRF1*, highlighting read coverages in the fertile line G398 and the sterile line G390. The positions are mapped relative to a 2-kb flank sequence of *Asa4G04832* on the garlic genome, delineating the gene structure with two introns and three exons.

Supplementary Figure 4 Alignments of amplified sequences by the InDel marker *AsaNRF1*-M281 in the male-sterile line G390 and the fertile line G398.
