# Supplementary figures and images for: Identification of male-fertility gene AsaNRF1 and molecular marker development in cultivated garlic (Allium sativum L.)
Source: Front Plant Sci. 2024 May 28;15:1419260. doi: 10.3389/fpls.2024.1419260 (PMC11165202; doi:10.3389/fpls.2024.1419260)

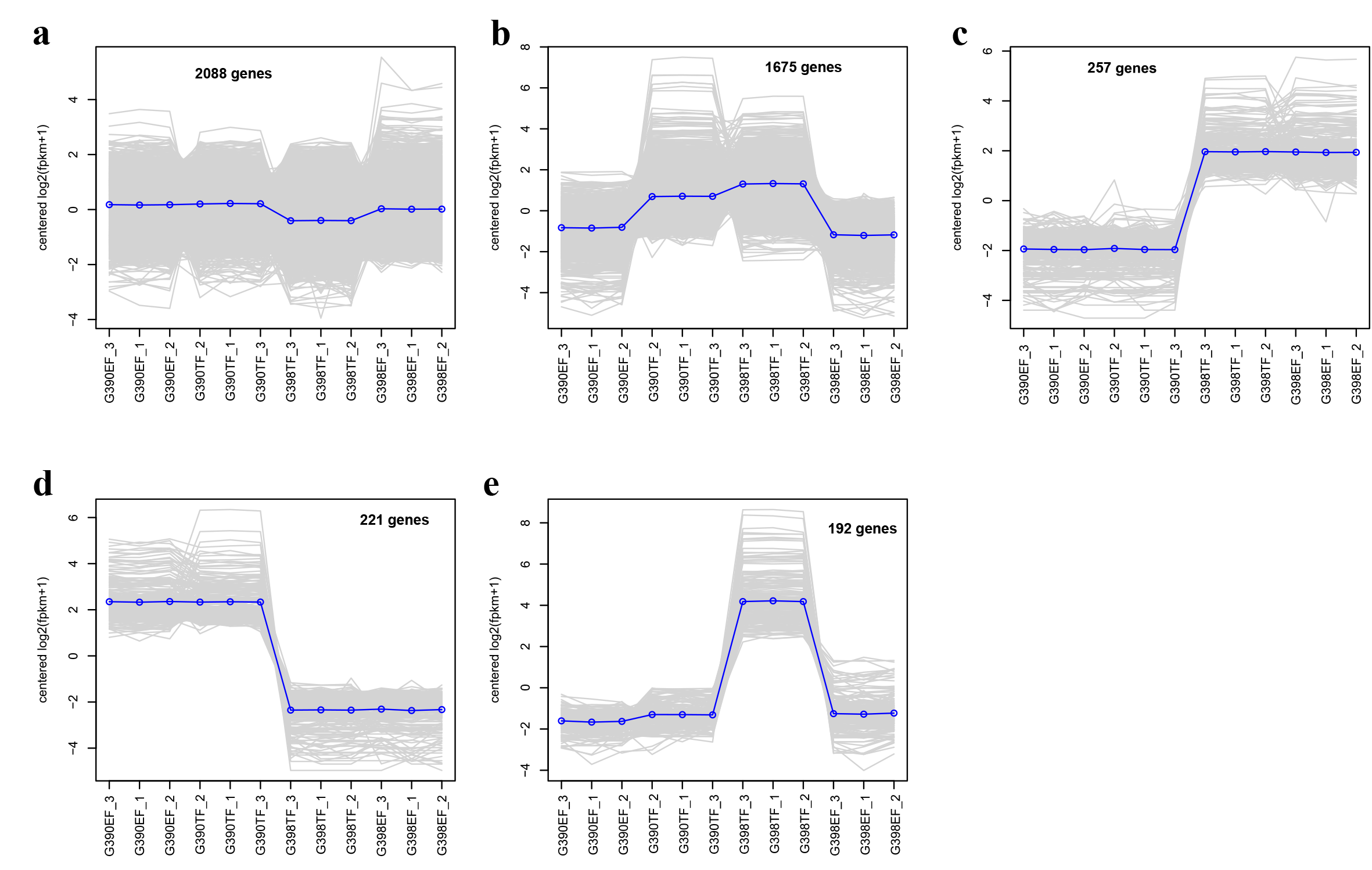

Supplement: Supplementary Figure 1 — K-means clustering of differentially expressed genes. (A) Non-prefer-expressed DEGs between G398 and G390. (B) Genes both highly expressed in G398TF and G390TF (median value of foldchange < 2). (C) Genes that were specific highly preferentially expressed in G398. (D) Genes that were specific highly preferentially expressed in G390. (E) Genes that were specific highly preferentially expressed in the tetrad-stage flower buds of the fertile line G398. [file Image_1.tif]

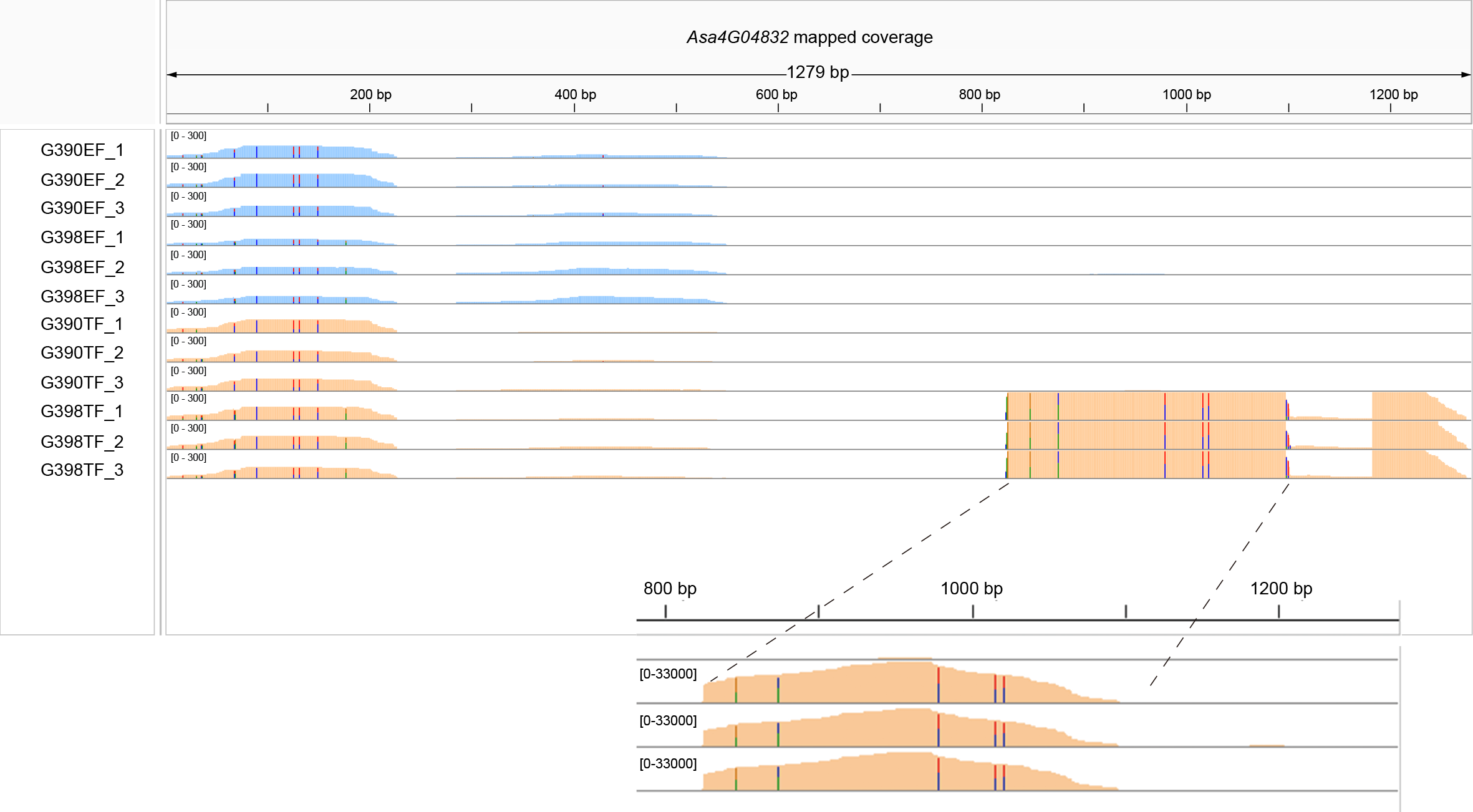

Supplement: Supplementary Figure 2 — RNA-Seq read-mapping pattern of the callose degradation homologous gene Asa4G04832. Read coverages of Asa4G04832 in the fertile line G398 and the sterile line G390 are shown. Positions are relative to the Asa4G04832 start codon. [file Image_2.tif]

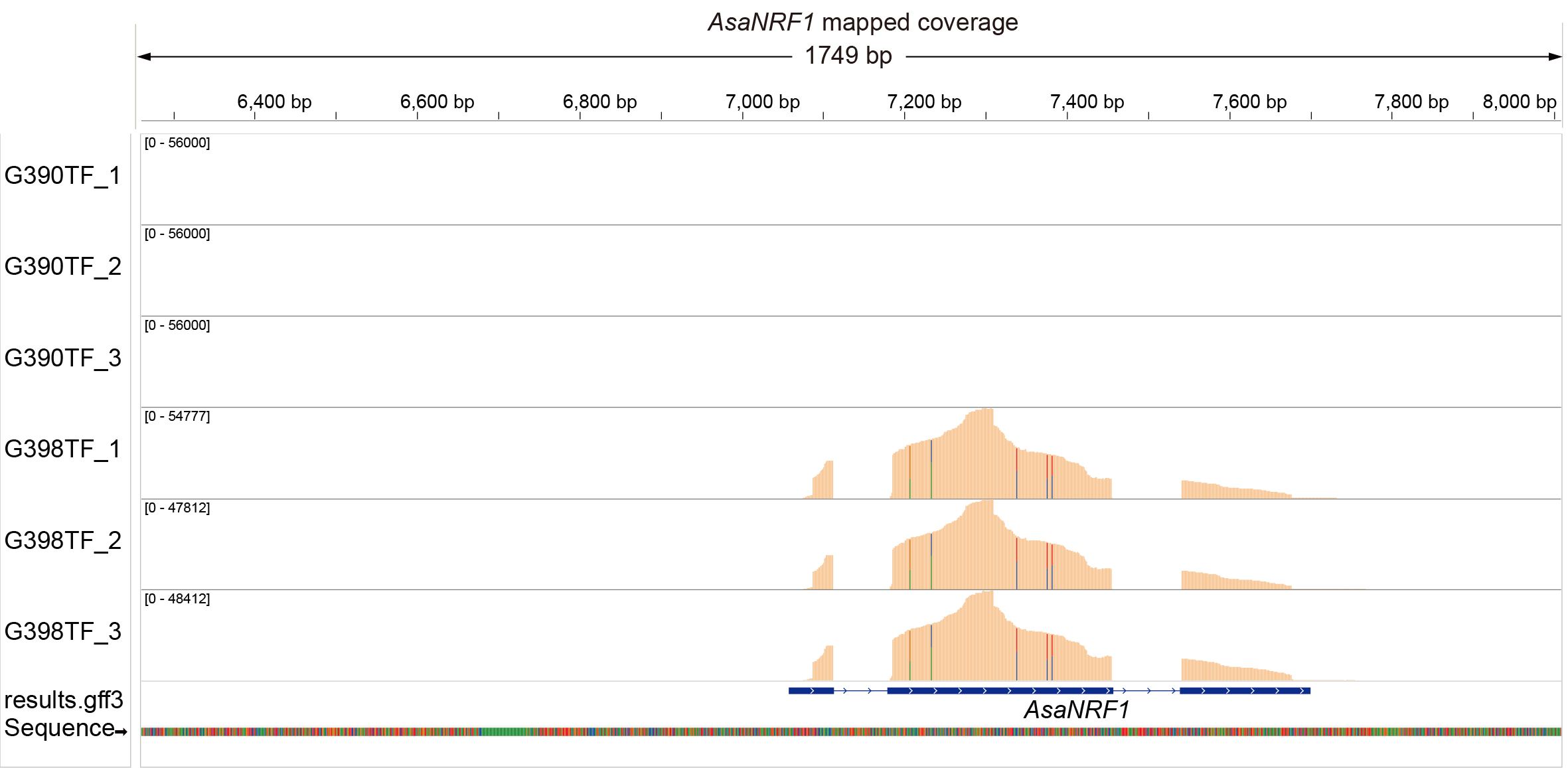

Supplement: Supplementary Figure 3 — RNA-Seq read-mapping pattern of AsaNRF1, highlighting read coverages in the fertile line G398 and the sterile line G390. The positions are mapped relative to a 2-kb flank sequence of Asa4G04832 on the garlic genome, delineating the gene structure with two introns and three exons. [file Image_3.tif]

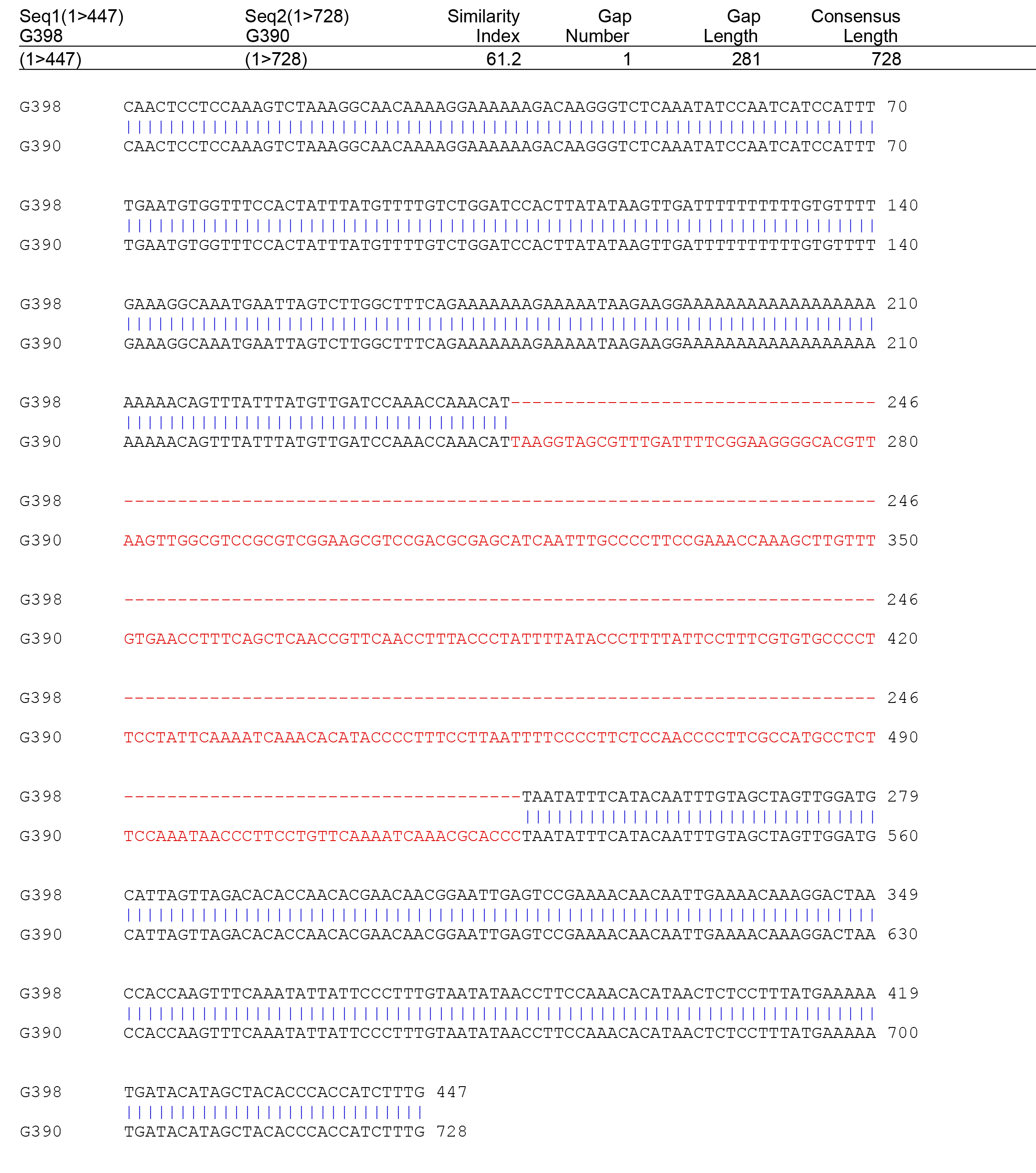

Supplement: Supplementary Figure 4 — Alignments of amplified sequences by the InDel marker AsaNRF1-M281 in the male-sterile line G390 and the fertile line G398. [file Image_4.tif]
